# Increased Risk of Alzheimer's Disease With Glycemic Variability: A Systematic Review and Meta-Analysis

**DOI:** 10.7759/cureus.73353

**Published:** 2024-11-09

**Authors:** Paul Nichol G Gonzales, Encarnita R Ampil, Joseree-Ann S Catindig-Dela Rosa, Steven G Villaraza, Ma. Lourdes C Joson

**Affiliations:** 1 Department of Neurology, Jose R. Reyes Memorial Medical Center, Manila, PHL; 2 Department of Neuroscience and Behavioral Medicine, University of Santo Tomas Hospital, Manila, PHL; 3 Faculty of Medicine and Surgery, University of Santo Tomas, Manila, PHL; 4 Institute for Neurosciences, St. Luke's Medical Center - Global City, Taguig, PHL

**Keywords:** alzheimer disease, blood glucose fluctuations, cognitive decline, cognitive impairment, dementia, fluctuating glucose level, glycemic variability

## Abstract

There is increasing evidence that establishes a connection between fluctuations in glucose metabolism and the onset of Alzheimer's disease (AD). Current research supports the notion that this metabolic imbalance significantly affects cognitive health. However, the specific mechanisms through which these fluctuations influence neurodegeneration, eventually leading to AD, require further exploration. This study aims to determine the risk of AD among individuals with fluctuations in blood glucose levels, with or without type 2 diabetes mellitus (T2DM), further providing the most recent and thorough overview of the evidence in this area. Following the Preferred Reporting Items for Systematic Reviews and Meta-Analyses (PRISMA) 2020 guidelines, a thorough search was carried out utilizing particular phrases in the PubMed, Elsevier, Research Gate, and Cochrane databases: (“glucose variability” or “glycemic variability” or “glucose fluctuation” or “glucose instability” or “glycemic fluctuation”) and (“Alzheimer’s disease” or “Alzheimer disease” or “Alzheimer dementia” or “Alzheimer”). Studies published between January 2014 to January 2024, written in English, and examining the relationship between glucose variability and AD, were included. The outcomes measured were risk of cognitive impairment and AD, cognitive performance, and risk of AD. The results of the literature search produced 142 records, with six studies meeting the eligibility criteria. Parameters for glycemic variability included fasting plasma glucose (FPG) variability, glycated hemoglobin (HbA1c) variability, FPG variability independent of the mean (VIM), FPG coefficient of variation (CV), and FPG standard deviation (SD). The studies revealed a positive correlation between glycemic variability and the risk of AD over time, and the findings indicated that maintaining stable glycemic levels may reduce the risk of cognitive decline among individuals with or without T2DM. Due to the small number of studies that are currently available, despite a calculated relative risk of 2.65 indicating a higher risk of AD among subjects with glycemic variability, the inclusion of the null value in the confidence interval (0.61-11.45) renders these findings not statistically significant. This comprehensive review demonstrated that, in people with or without diabetes, glycemic variability influences cognitive decline and the risk of AD. The studies demonstrated a correlation between higher fluctuations in glucose levels and an increased risk of AD, highlighting the importance of managing blood sugar levels to mitigate dementia risks. Despite these strong associations, the actual incidence rates of AD in the studied populations remained relatively low. Overall, the results were not statistically significant. Further research is recommended to explore the risk of AD among individuals with fluctuations in blood glucose levels.

## Introduction and background

The aging population worldwide is expected to bring a significant increase in dementia prevalence, making this condition a global public health concern. It has an impact on families by raising the need for care, decreasing quality of life, and placing a significant financial strain on society. Furthermore, the majority of nations and territories continue to have high rates of sickness, indicating that increased awareness of detection could be a useful strategy for both prevention and treatment [1].

Type 2 diabetes mellitus (T2DM), being a recognized risk factor for Alzheimer's disease (AD), promotes mechanisms such as vascular damage, metabolic dysfunction, and blood-brain barrier disruption [2]. It accelerates the AD process by exacerbating key pathological mechanisms, which include increased production and decreased clearance of amyloid-β (Aβ), acceleration of tau-related changes through mechanisms such as altered insulin signaling, and increased vascular damage, and changes in cerebral blood flow that can contribute to brain pathologies and cognitive deficits associated with AD [3]. Most notably, T2DM promotes AD by influencing fluctuation in blood glucose levels, which causes increased oxidative stress and inflammation, leading to neuronal damage and impaired cognitive functions [4]. The effect of glucose in the brain is supported by the fact that its fluctuation has been shown to raise the risk of AD and cognitive impairment in people without T2DM [5]. According to human studies, insulin and insulin-like growth factor (IGF) signaling are significantly disrupted in AD, which supports the hypothesis that AD is a brain-specific form of "type 3 diabetes” [6].

The degree of glucose changes that take place within a certain time frame is known as glucose variability. Since glucose is the sole energy source available to the brain, problems with glucose metabolism are most likely to affect it [7]. The hypothesis that variability in glucose levels contributes to cognitive decline has gained substantial support through various studies [4,8-10]. Currently, the association of the fluctuations in glucose and AD has been explored, but a relationship has not been clearly elucidated. Hence, this study aims to determine the risk of AD among individuals with fluctuations in blood glucose levels, with or without T2DM.

## Review

Methods

This study adhered to the Preferred Reporting Items for Systematic Reviews and Meta-Analyses (PRISMA) guidelines to ensure a thorough literature search. A systematic review and meta-analysis approach was followed using step-by-step guidance. Studies from PubMed, Elsevier, Research Gate, and Cochrane journals were searched with the following terms: (“glucose variability” or “glycemic variability” or “glucose fluctuation” or “glucose instability” or “glycemic fluctuation”) and (“Alzheimer’s disease” or “Alzheimer disease” or “Alzheimer dementia” or “Alzheimer”).

The inclusion criteria for this review considered studies that were published from January 2014 to January 2024 in the English language, studies that examined the relationship of glucose variability with AD regardless of diabetes status, and studies where the full manuscript is available. Outcome measures included any of the following: risk of AD, cognitive performance, and incidence of AD. Studies meeting the following conditions were excluded: duplicate articles, animal studies, drug trials, editorials, letters to the editor, recommendations, monographs, dissertations, and theses were excluded, as were articles that did not require a diagnosis of AD.

After eliminating duplicates, the studies from the initial search were assessed for eligibility based on their titles and abstracts by two authors (PNG and JCD). Any studies that did not align with the review's objectives were excluded. A second screening round was conducted by two authors (ERA and MCJ) using the complete texts in which inconclusive studies or those with insufficient information concerning glycemic variability with AD, or where the outcome cannot be directly extracted, were excluded. References identified during the second screening underwent another evaluation by another author (SGV). The final selection of studies for the systematic review and meta-analysis was determined afterward.

Data items extracted from the studies included the following: author, origin, year of publication, study design, population used, study intervention or exposure, outcome measure, cognitive tests used, and findings.

To evaluate the risk of bias in the studies, the authors employed the Newcastle-Ottawa Quality Assessment Scale from the study by Stang, which is widely used for observational studies [11]. This scale employs a "star" rating system, evaluating eight criteria grouped into three main facets: (1) selection of study groups, (2) comparability of groups based on design or analyses, and (3) ascertainment of exposure for case-control studies or outcome for cohort studies. A maximum of one star is awarded for each item, except for the item on comparability, which allows up to two stars. A total of nine stars can be allotted, indicating top quality. The risk of bias was determined by the number of stars awarded: high risk with zero to three stars, medium risk with four to six stars, and low risk with seven to nine stars. For cross-sectional studies, an adapted version of the Newcastle-Ottawa Assessment Scale from the study by Herzog et al. was utilized, with a total allowable score of 10 [12].

Results

Findings From the Literature Search

**Figure 1:**
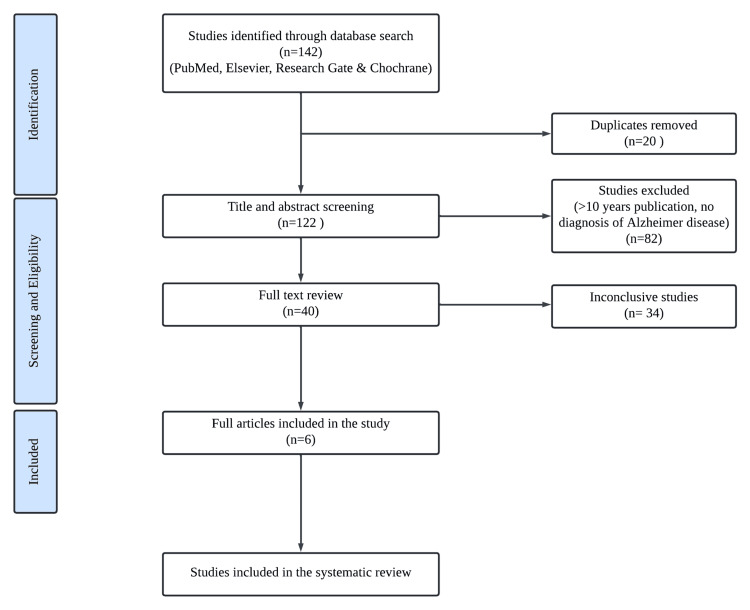
PRISMA flow diagram PRISMA: Preferred Reporting Items for Systematic Reviews and Meta-Analyses

A total of 142 records were obtained after a literature search in PubMed, Elsevier, Research Gate, and Cochrane. There were 20 duplicate studies removed. Of the 122 remaining studies, 82 were excluded due to a publication date of 10 years or more and if there is no diagnosis of AD. Out of the 40 full-text papers that were included, 34 studies were deemed inconclusive due to studies with contradicting conclusions, insufficient follow-up periods, or a limited number of participants, which can affect reliability and generalizability. Subsequently, all six articles satisfied the eligibility criteria and were incorporated into the study. The procedure employed in selecting the studies for the review is shown in Figure 1.

Features of the Studies Included

The six included studies [9,13-17] were published between July 2017 and July 2023, with sample sizes ranging from 80 to three million participants, all with a mean age above 60 years. Variability in glucose levels was derived from either fasting plasma glucose or HbA1c levels. See Table 1 for the characteristics of the studies.

**Table 1:** Studies included in the systematic review Source: Refs. [9,13-17] AD - Alzheimer's disease; VaD - Vascular dementia; MMSE - Mini-Mental State Examination; MoCA - Montreal Cognitive Assessment; ADAS-Cog - Alzheimer’s Disease Assessment Scale-Cognitive Subscale; CDR - Clinical Dementia Rating; FPG - Fasting Plasma Glucose; FG - Fasting Glucose; HbA1c - Hemoglobin A1c; VIM - Variability Independent of the Mean; rCMRgl - Cerebral Metabolic Rate for Glucose; AVLT-LTM - Auditory Verbal Learning Test Long-Term Memory Score, CFT-R - Complex Figure Test Recall, COWAT - Controlled Oral Word Association Test; MTA - Medial Temporal Atrophy; FDG-PET - Fludeoxyglucose-18 (FDG) Positron Emission Tomography; BMI - Body Mass Index

Author/Year	Study Design	Study Population	Intervention/Exposure	Outcome Measure	Cognitive Tests	Findings
Li et al. [13], 2017 Taiwan	Cohort study	Diabetic cognitively intact individuals	Visit-to-visit variations in FPG and HbA1c	Incidence of AD	Not stated	Glycemic variability is independently associated with Alzheimer disease in patients with type 2 diabetes
Lee et al. [14], 2022 Korea	Cohort study	Diabetic cognitively intact individuals	Fasting glucose variability [FG-VIM]	Risk of AD and other dementia	Mini-Mental State Examination [MMSE], Alzheimer’s Disease Assessment Scale-Cognitive Subscale [ADAS-Cog], Clinical Dementia Rating [CDR]	Increased fasting glucose variability predicts a higher risk of dementia in individuals with diabetes.
Wium-Andersen et al. [9] 2019, Denmark	Cohort study	Patients with type 1 or type 2 diabetes and individuals with intact cognition	Diabetes diagnosis, HbA1c levels	Risk of AD and other dementia, cognitive performance	Intelligenz-Struktur-Test	Diabetes is associated with an increased risk of dementia; the impact of high HbA1c on dementia risk varies.
Zhang et al. [16] 2023, China	Cross-sectional	Cognitively impaired individuals regardless of diabetic status	Glycemic variability [VIM]	Risk of AD through medial temporal atrophy [MTA]	MMSE, Montreal Cognitive Assessment [MoCA]	Glycemic variability, as indicated by VIM, is related to reduced cognitive function and non-linearly to MTA scores.
Burns et al. [17] 2018, USA	Cohort study	Cognitively normal adults with no diabetes	Longitudinal changes in serum glucose levels	Risk of AD by changes in cerebral metabolic rate for glucose [rCMRgl] in AD-related brain regions measured by FDG-PET	Auditory Verbal Learning Test Long Term Memory score [AVLT-LTM], Complex Figure Test Recall [CFT-R], Controlled Oral Word Association Test [COWAT]	Increasing serum glucose levels were significantly associated with declines in rCMRgl in key brain regions affected by Alzheimer’s disease, independent of APOE ε4 status.
Lee et al. [15] 2018, Korea	Cohort study	Adults without dementia, hypertension, diabetes, or dyslipidemia	Visit-to-visit variability in blood pressure, glucose, cholesterol, and BMI	Incident dementia categorized as AD or VaD	Not stated	Higher variability in blood pressure, glucose, cholesterol, and BMI was associated with an increased risk of future dementia, particularly AD and VaD.

Risk of Bias Assessment

The assessment of study quality by the Newcastle-Ottawa Quality Assessment Scale and the adapted version indicated that all the included studies were of high quality, which implied minimal selection bias and comparable cohorts (Table 2).

**Table 2:** Risk of bias using the Newcastle-Ottawa Assessment Scale for cohorts and the adapted version for cross-sectional studies Source: Refs. [9,13-17]

	Selection [up to 1 star for each]								Comparability (up to 2 stars)		Outcome (up to 1 star for each)					
Author (Cohort)	Representativeness of the exposed cohort		Selection of the non-exposed cohort		Ascertainment of exposure		Demonstration that outcome of interest was not present at start of study		Comparability of cohorts on the basis of the design or analysis		Assessment of outcome		Was follow-up long enough for outcomes to occur		Adequacy of follow up of cohorts	Overall quality, ★/9
Li et al. [13] 2017	★		★		★		★		★★		★		★		★	9/9
Lee et al. [14] 2022	★		★		★		★		★★		★		★		★	9/9
Wium-Andersen et al. [9] 2019	★		★		★		★		★★		★		★		★	9/9
Lee et al. [15] 2018	★		★		★		★		★★		★		★		★	9/9
Burns et al. [17] 2018	★		★		★		★		★★		★		★		★	9/9
		Selection								Comparability		Outcome				Total
Author (Cross-sectional)		Representativeness of the sample (up to 1 star)		Sample size (up to 1 star)		Nonrespondents (up to 1 star)		Ascertainment of exposure (up to 2 stars)		Based on design and analysis (up to 2 stars)		Assessment of outcome (up to 2 stars)		Statistical Test (up to 1 star)		★/10
Zhang et al. [16] 2023		★		★		★		★★		★★		★★		★		10/10

Meta-Analysis of Glycemic Variability on AD

**Figure 2:**
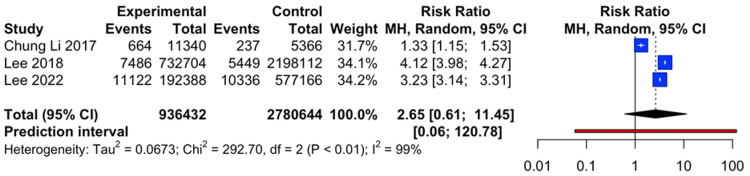
Forest plot Source: Refs. [13-15]

Figure 2 illustrates studies comparing glycemic variability and AD, with sufficient data to generate a forest plot (three out of six studies). The forest plot revealed significant statistical heterogeneity among the studies, indicating non-homogeneity when using the fixed effects model (chi² = 292.70, P < 0.01, I² = 99%). With a total sample size of 3,717,076 across all studies, the incidence rate of AD in subjects with glycemic variability was 2.06%, compared to 0.58% in those without glycemic variability. This resulted in a relative risk of 2.65, indicating that individuals with glycemic variability had a 2.65 times higher risk of developing AD than those with normal glucose levels. The 95% confidence interval for the relative risk ranged from 0.61 to 11.45. Since the confidence interval included the null value of one, the findings were not statistically significant, suggesting no significant difference in AD incidence rates between patients with and without glycemic variability.

Discussion

Glycemic variability could hasten the negative consequences of long-term ambient hyperglycemia [7]. Although it holds significant clinical value, the association between glucose variability and AD, regardless of diabetes status, has not been thoroughly investigated. In this systematic review and meta-analysis, only six studies in compliance with the inclusion and exclusion criteria were included. The findings demonstrated that markers of blood glucose fluctuation, including FPG variability, and glycated hemoglobin (HbA1c) variability, were closely associated with cognitive dysfunction, specifically among those with AD. Furthermore, glucose variability has been associated with structural and metabolic processes in AD.

Glycemic Variability Increases the Risk of AD

The connection between glycemic fluctuation and cognitive decline, especially the risk of AD, has received more attention in recent research. For instance, the study by Li et al., over a median follow-up of 8.88 years involving 16,706 patients aged 60 and above with T2DM but without AD diagnosis, showed that higher variability in FPG and HbA1c - measured as the ratio of the standard deviation to the mean of FPG or HbA1c levels over multiple visits within a specific time period - significantly predicted the risk of developing AD (P < 0.05). After adjusting for various co-factors, the study found that there was a high likelihood of developing AD in people with the highest fluctuations in their FPG variability (HR = 1.27; 95% CI 1.06-1.52) and HbA1c variability (HR = 1.32; 95% CI 1.11-1.58) [13].

Fasting glucose variability, as measured by variability independent of the mean (FG-VIM), was another parameter for blood glucose fluctuations. A study by Lee et al. looked into a nationwide cohort involving 769,554 diabetic individuals using FG-VIM. Cognitive decline on follow-up was assessed using the Mini-Mental State Examination (MMSE), Alzheimer’s Disease Assessment Scale-Cognitive Subscale (ADAS-Cog), and Clinical Dementia Rating (CDR). According to the study, there was an 18% increased risk of all-cause dementia among those with T2DM who were in the highest quartile of fasting glucose variability (HR = 1.18; 95% CI: 1.15-1.21), a 19% higher risk of AD (HR = 1.19; 95% CI: 1.15-1.22), and a 17% higher risk of vascular dementia (VaD) (HR = 1.17; 95% CI: 1.09-1.25) [14]. This emphasized that FG-VIM over a five-year period can be used as a factor for dementia risk, including AD.

Diabetes mellitus, in general, is related to cognitive decline. Wium-Andersen et al. conducted a study that investigated 148,036 individuals with diabetes registered in various Danish health registers and cohort studies. They highlighted that both type one and type two diabetes and elevated HbA1c levels (≥48 mmol/mol, the diagnostic limit for diabetes) were associated with lower cognitive performance and an increased risk of dementia (HR = 1.94; 95% CI: 1.10-3.44). However, when looking into the risk of dementia, specifically AD, patients with type 1 diabetes had no increased risk of AD, while those with T2DM had an increased risk of AD (HR = 1.12; 95% CI: 1.04-1.21) [9].

Even in those without diabetes, variations in blood sugar levels were linked to changes in cognitive function [18]. A nationwide study by Lee et al. involved 2,930,816 subjects without a history of diabetes and investigated the effect of visit-to-visit variability in metabolic parameters, including glucose levels, on dementia risk, specifically AD. Participants were split into four groups (quartiles) based on the level of variability in blood sugar levels using the FPG coefficient of variation, FPG standard deviation, and FPG variability independent of the mean (VIM). The highest quartile (fourth quartile) of glucose variability had a 13% increased risk of developing AD (HR = 1.13; 95% CI: 1.09-1.17; P < 0.001) compared to the lowest quartile (first quartile). According to the study, even among non-diabetics, higher glucose fluctuation was linked to an increased risk of AD. It also suggested that preserving steady glucose levels throughout time may be crucial in lowering the risk of AD [16].

Structural and Metabolic Alterations in AD Are Associated with Glycemic Variability

A higher glycemic variability has been linked to poorer cognitive outcomes, which have been correlated with the presence and degree of medial temporal lobe atrophy (MTA). These indicators were used to diagnose and assess the progression of AD [19]. A study by Zhang et al. over a group of 461 cognitively impaired participants showed that, regardless of diabetes status, a higher glycemic variability, measured by VIM, was strongly linked to lower cognitive performance, as measured by MMSE (r = -0.729, P < 0.01] and MoCA scores (r = -0.710, P < 0.01), and more severe MTA using the MTA scoring on coronal T1-weighted MRI among patients with memory deficits (r = 0.498, P < 0.01). This study suggested the mechanism of glycemic variability for AD could be through atrophy of the medial temporal lobe [16].

When evaluated in vivo using fluorodeoxyglucose (F18) positron emission tomography (FDG-PET), AD was characterized by reduced cortical activity owing to neuronal death, especially in temporoparietal regions. It was believed that the decrease in activity in these locations was a result of neurodegenerative processes that impaired synaptic activity [17]. The study by Burns et al. included 80 non-diabetic adults with a mean age of 61.5 years and investigated whether variations in serum glucose levels across time were associated with changes in FDG-PET measurements of cerebral metabolic rate for glucose (rCMRgl) in brain regions affected in AD (i.e., temporoparietal regions, precuneus/posterior cingulate, precuneus/posterior cingulate). Key findings showed a statistically significant increase in serum glucose levels over time, from 91.0 ± 8.0 mg/dL to 95.2 ± 8.6 mg/dL (P < 0.001). Additionally, there was a strong negative relationship between scores in the complex figure test recall and variations in serum glucose levels (r = -0.3, P = 0.002), reflecting cognitive decline. There were notable inverse relationships between rises in blood glucose and falls in rCMRgl, especially in the prefrontal and temporoparietal regions and bilaterally in the precuneus and posterior cingulate areas (P < 0.05, corrected for multiple comparisons), implicating the relationship of longitudinal changes in serum glucose levels with hypometabolism in regions linked to AD [20].

The associations between serum glucose variability, brain atrophy, and cerebral hypometabolism in AD suggest a close relationship between metabolic changes, and structural and functional impairment in the brain. As brain tissue shrinks and neurons are lost, the affected regions become less metabolically active [21]. This decline in metabolic activity reflects the underlying neurodegenerative processes occurring in AD, where the medial temporal lobe exhibits the greatest atrophy and is the first site of AD pathogenesis [22].

Across all studies, there was a consistent observation that, despite glucose levels, elevated glycemic variability was associated with AD. Li et al., Lee et al., Wium-Andersen et al., and Lee et al. discussed the implications of glycemic variability on cognitive performance and AD risk [9,13-15]. Li et al. highlighted that FPG and HbA1c visit-to-visit changes were substantially linked to a higher risk of AD in T2DM patients [13]. Similarly, Lee et al. found that fasting glucose variability over several months to years was independently associated with AD, suggesting that glucose stability might play a role in cognitive health in diabetes [14]. Further, Wium-Andersen et al. found an increased risk of AD despite diabetes type [9]. Additionally, Lee et al. demonstrated that high variability in metabolic parameters such as glucose significantly raises the risk of all dementia types, including AD, suggesting that stabilizing such parameters may reduce AD risk [15].

Burns et al. and Zhang et al. both focused on how diabetes-related metabolic changes affected the brain regions commonly impacted in AD [16,17]. Burns et al. showed that changes in the metabolism of brain regions relevant to AD as shown by PET scans were correlated with long-term increases in blood glucose levels [16]. Zhang et al. added that glycemic variability was associated with a reduction in cognitive function and MTA, suggesting a direct link between blood glucose control and brain structural changes suggestive of AD [17].

This systematic review and meta-analysis collectively highlight a significant association between glucose fluctuations and AD. Despite these strong associations, the actual incidence rates of AD in the studied populations remain relatively low. One possible explanation for the low incidence of AD despite this association might be related to the multifactorial nature of AD, where other factors such as genetics, lifestyle, and environmental influences play crucial roles. Moreover, it could be that, while glycemic variability influences the risk, it does so in conjunction with these other factors, and perhaps only becomes a dominant risk factor when combined with certain genetic profiles or other predisposing conditions [23].

The effective management of glycemic levels in clinical settings, especially in patients identified as high risk, might also be contributing to lower-than-expected AD incidences in populations where diabetes is well-managed. While the association between glycemic variability and AD is strong, the actual manifestation of AD might be mitigated or influenced by several other factors, which helps explain the relatively low incidence of AD in the face of this significant association. These findings imply the complexity of AD as a disease and the need for holistic approaches to its prediction, prevention, and management.

Strengths and Limitations

This systematic review and meta-analysis emphasized the increased risk of AD with glycemic variability regardless of diabetes status. The studies involved have lengthy follow-up periods and large cohorts, allowing for the observation of long-term trends and generalizability in the relationship between glycemic variability and cognitive decline. Additionally, the studies reviewed were specifically associated with AD. However, several limitations were identified. One notable limitation was the limited number of studies available. Despite an extensive database search, most research on glycemic variability focused on the risk of cognitive decline and dementia in general. Studies addressing the risk of AD in relation to glycemic variability still need to be developed, pointing to potential directions for future research. Another limitation was the reliance on observational studies, which cannot definitively establish causality despite including data from longitudinal follow-ups. Additionally, the focus on older adults may overlook the potential effects of glycemic variability in the younger age group. Lastly, the statistical heterogeneity in this systematic review made the meta-analysis challenging due to the variability in results among the included studies. This limitation can hinder the synthesis of evidence and may make it difficult to draw definitive conclusions or identify consistent trends across the studies.

## Conclusions

This comprehensive review demonstrated that, in people with or without diabetes, glycemic variability influences cognitive decline and the risk of AD. Individually, the studies showed a link between larger fluctuations in glucose levels and a higher risk of AD, suggesting that managing blood sugar could help reduce the risk of dementia. However, while these individual findings were statistically significant on their own, the overall results from the meta-analysis were not, due to the high heterogeneity of the studies. Further research is recommended to explore the risk of AD among individuals with fluctuations in blood glucose levels.

## References

[1] Li X, Feng X, Sun X, Hou N, Han F, Liu Y (2022). Global, regional, and national burden of Alzheimer's disease and other dementias, 1990-2019. Front Aging Neurosci.

[2] Biessels GJ, Despa F (2018). Cognitive decline and dementia in diabetes mellitus: mechanisms and clinical implications. Nat Rev Endocrinol.

[3] Baglietto-Vargas D, Shi J, Yaeger DM, Ager R, LaFerla FM (2016). Diabetes and Alzheimer's disease crosstalk. Neurosci Biobehav Rev.

[4] Ding J, Shi Q, Tao Q, Su H, Du Y, Pan T, Zhong X (2023). Correlation between long-term glycemic variability and cognitive function in middle-aged and elderly patients with type 2 diabetes mellitus: a retrospective study. PeerJ.

[5] Bancks MP, Carnethon MR, Jacobs DR Jr (2018). Fasting glucose variability in young adulthood and cognitive function in middle age: the coronary artery risk development in young adults (CARDIA) study. Diabetes Care.

[6] de la Monte SM, Wands JR (2008). Alzheimer's disease is type 3 diabetes-evidence reviewed. J Diabetes Sci Technol.

[7] Chi H, Song M, Zhang J, Zhou J, Liu D (2023). Relationship between acute glucose variability and cognitive decline in type 2 diabetes: a systematic review and meta-analysis. PLoS One.

[8] Rawlings AM, Sharrett AR, Mosley TH, Ballew SH, Deal JA, Selvin E (2017). Glucose peaks and the risk of dementia and 20-year cognitive decline. Diabetes Care.

[9] Wium-Andersen IK, Rungby J, Jørgensen MB, Sandbæk A, Osler M, Wium-Andersen MK (2019). Risk of dementia and cognitive dysfunction in individuals with diabetes or elevated blood glucose. Epidemiol Psychiatr Sci.

[10] Zheng B, Su B, Price G, Tzoulaki I, Ahmadi-Abhari S, Middleton L (2021). Glycemic control, diabetic complications, and risk of dementia in patients with diabetes: results from a large U.K. cohort study. Diabetes Care.

[11] Stang A (2010). Critical evaluation of the Newcastle-Ottawa scale for the assessment of the quality of nonrandomized studies in meta-analyses. Eur J Epidemiol.

[12] Herzog R, Álvarez-Pasquin MJ, Díaz C, Del Barrio JL, Estrada JM, Gil Á (2013). Are healthcare workers' intentions to vaccinate related to their knowledge, beliefs and attitudes? A systematic review. BMC Public Health.

[13] Li TC, Yang CP, Tseng ST (2017). Visit-to-visit variations in fasting plasma glucose and HbA1c associated with an increased risk of Alzheimer disease: Taiwan diabetes study. Diabetes Care.

[14] Lee DY, Kim J, Park S (2022). Fasting glucose variability and the risk of dementia in individuals with diabetes: a nationwide cohort study. Diabetes Metab J.

[15] Lee SH, Han K, Cho H (2018). Variability in metabolic parameters and risk of dementia: a nationwide population-based study. Alzheimers Res Ther.

[16] Zhang S, Wang A, Liu S, Liu H, Zhu W, Zhang Z (2023). Glycemic variability correlates with medial temporal lobe atrophy and decreased cognitive performance in patients with memory deficits. Front Aging Neurosci.

[17] Burns CM, Kaszniak AW, Chen K, Lee W, Bandy DJ, Caselli RJ, Reiman EM (2018). Longitudinal changes in serum glucose levels are associated with metabolic changes in Alzheimer’s disease related brain regions. J Alzheimers Dis.

[18] Ravona-Springer R, Moshier E, Schmeidler J (2012). Changes in glycemic control are associated with changes in cognition in non-diabetic elderly. J Alzheimers Dis.

[19] Scheltens P, Leys D, Barkhof F (1992). Atrophy of medial temporal lobes on MRI in "probable" Alzheimer's disease and normal ageing: diagnostic value and neuropsychological correlates. J Neurol Neurosurg Psychiatry.

[20] Adams JN, Lockhart SN, Li L, Jagust WJ (2019). Relationships between tau and glucose metabolism reflect Alzheimer’s disease pathology in cognitively normal older adults. Cereb Cortex.

[21] Strom A, Iaccarino L, Edwards L (2022). Cortical hypometabolism reflects local atrophy and tau pathology in symptomatic Alzheimer's disease. Brain.

[22] Braak H, Braak E (1991). Neuropathological stageing of Alzheimer-related changes. Acta Neuropathol.

[23] Van Cauwenberghe C, Van Broeckhoven C, Sleegers K (2016). The genetic landscape of Alzheimer disease: clinical implications and perspectives. Genet Med.

